# Influence of alkali metals (Na, Li, Rb) on the performance of electrostatic spray-assisted vapor deposited Cu_2_ZnSn(S,Se)_4_ solar cells

**DOI:** 10.1038/srep22109

**Published:** 2016-02-26

**Authors:** Giovanni Altamura, Mingqing Wang, Kwang-Leong Choy

**Affiliations:** 1 UCL Institute for Materials Discovery, University College London, Roberts Building, Malet Place, London WC1E7JE, United Kingdom

## Abstract

Electrostatic Spray-Assisted Vapor Deposition (ESAVD) is a non-vacuum and cost-effective method to deposit metal oxide, various sulphide and chalcogenide at large scale. In this work, ESAVD was used to deposit Cu_2_ZnSn(S_1−x_Se_x_)_4_ (CZTSSe) absorber. Different alkali metals like Na, Li and Rb were incorporated in CZTSSe compounds to further improve the photovoltaic performances of related devices. In addition, to the best of our knowledge, no experimental study has been carried out to test the effect of Li and Rb incorporation in CZTSSe solar cells. X-ray diffraction, Raman spectroscopy, scanning electron microscopy, and glow discharge spectroscopy have been used to characterize the phase purity, morphology and composition of as-deposited CZTSSe thin films. Photovoltaic properties of the resulting devices were determined by completing the solar cells as follows: Mo/CZTSSe/CdS/i-ZnO/Al:ZnO/Ni/Al. The results showed that Li, Na and Rb incorporation can increase power conversion efficiency of CZTS devices up to 5.5%. The introduction of a thiourea treatment, has improved the quality of the absorber^|^buffer interface, pushed the device efficiency up to 6.3% which is at the moment the best reported result for ESAVD deposited CZTSSe solar cells.

Due to the extremely low raw material costs, earth abundant, sustainability and non-toxicity, kesterite Cu_2_ZnSn(S_1−x_Se_x_)_4_ (CZTSSe) compounds can play a major role in future photovoltaic technologies. This key aspect related to their optical properties–bandgap range from 1.0 eV (Cu_2_ZnSnSe_4_) to 1.5 eV (Cu_2_ZnSnS_4_) and absorption coefficient higher than 10^4 ^cm^−1^ –makes possible to foresee a photovoltaic thin film technology scalable at TW/year. Thus many groups have been focused on elaborating such materials in the past few years, using selenization of vacuum deposited precursors^1^, co-evaporation of the different elements followed by annealing^2^, or a hydrazine-based solution processing leading to a record conversion efficiency of 12.7%^3^. Indeed the latter record presents an inconvenience: hydrazine is a relatively toxic and dangerous solvent, which during the years has limited the mass production of solar cells by this method. In order to overcome this problem scientific community has focused on other non-vacuum deposition techniques. Guo *et al.* have reported the synthesis of stoichiometric Cu_2_ZnSnS_4_ (CZTS) nanocrystals and showed that the efficiency of derived solar cells can reach as high as 7.2%^4^. Nanocrystals deposition method normally involves complex chemical synthesis and purification process, which is also rather challenging to be scaled up at industrial level. Hillhouse’s group have developed a new solution that yields stable inks composed of molecular complexes in a dimethyl sulfoxide (DMSO)/thiourea solution achieving 11% efficiency^5^. In all of the reported high efficiency non-vacuum deposited kesterite solar cells, multilayer spin coating was chosen to deposit the absorber to achieve the desired thickness. Spin coating is a widely used technique for lab use, but it is not suitable for scale up in industry due to its high material consumption, long processing times and the restriction to small-area.

Electrostatic Spray-Assisted Vapor Deposition (ESAVD) is a non-vacuum process in which a mixture of chemical precursors is atomized to form aerosol. The aerosol is charged and directed towards a heated substrate where it would undergo decomposition and chemical reaction to deposit a stable solid film onto the substrate. ESAVD is a scalable process at industrial level and it can be operated in open air. It is also very easy to be adapted to large area deposition using multiple spray atomizers with the deposition uniformity as high as 90%. ESAVD has demonstrated to be suitable for non-vacuum deposition of Cu(In,Ga)Se_2_ (CIGS) and CZTSSe^6^ as long as other chalcogenides^7^. The setup of ESAVD technique to deposit CZTSSe compounds for this work is already described in ref. 6.

The beneficial effect for photovoltaic properties coming from the incorporation of alkali metals like sodium^8^ in chalcogenide-based thin film solar cells has already been widely demonstrated^9^. It mainly improves device performance through the increase of FF, Voc and hole density^9^. Although the exact role of Na on the structural and electronic properties of the absorber material is not clearly understood, defect passivation at the grain boundaries is nowadays the most considered explanation^9^. In the case of CZTSSe, it has been demonstrated that Na can mainly improve open-circuit voltage (Voc) and fill-factor (FF)^10^. To the best of our knowledge, other alkali materials like Li and Rb have never been incorporated in CZTSSe absorbers, therefore, that their possible implications in the absorber quality are unknown. This paper reports in details the characterization methods and experimental results of the CZTSSe layer incorporated with different alkali-content. Results are discussed and evaluated thereafter.

## Materials & Methods

### Substrate preparation

A 550 nm Mo coated-soda lime glass (R_□ _= 0.60 Ω/**□**), deoxidized for 10 s in a 10% NH3 solution, was used as the standard substrate. 10 nm of i-ZnO was deposited onto the Mo substrate by magnetron sputtering deposition (HHV 500 Auto vacuum coater, 1.0 W/cm^2^, 10^−3 ^mbar, 6.0 sccm Ar and 1.7 sccm O_2_, room temperature, 3 mins), using 99.99% i-ZnO Kurt Lesker purity targets. The thickness of the as-deposited ZnO monolayer was determined by Bruker Dektak XT Contact Profilometer with a 5% absolute accuracy.

### CZTS precursor preparation

In this article, an environmental friendly and sustainable ESAVD process has been exploited and developed for the synthesis of CZTSSe absorber layers^7^. In order to deposit CZTS film, a mixture of precursor solution containing copper acetate (Cu(OAc)_2_; ≥97.9%), zinc chloride (ZnCl_2_; ≥98.9%), tin chloride pentahydrate (SnCl_4_·5H_2_O; 98.9%), and thiourea (SC-(NH_2_)_2_; 99.0%) was dissolved in Dimethyl sulfoxide solvent and the deposition was performed at a temperature range of 250–450 °C. The composition of the precursor measured at X-ray fluorescence is meant to give a Zn-rich ([Zn]/[Sn] ~ 1.12) and Cu-poor ([Cu]/[Sn+Zn] + Zn ~ 0.73) absorber which is proved to deliver the best photovoltaic efficiency at the device level^2^. A solution of containing individually sodium chloride (NaCl, ≥99.9%), lithium chloride (LiCl, ≥99.0%) and rubidium chloride (RbCl, ≥99.0%) dissolved in deionized water is synthesized to incorporate different doping material into CZTS. The molarity of each element-chloride has been varied in the range 0.1−0.4 M with a step of 0.1 M. The as-deposited CZTS, before selenization annealing, has been soaked in the prepared alkali solution for a time range of 20, 40 and 60 minutes. Samples subjected to Li, Rb, and Na incorporation were labeled as A, B, and C, respectively as shown in Table 1.

### Selenization

Larramona *et al.*^11^ has demonstrated that the use of a hot plate in a N_2_ glove box, to compact the precursor layer, allows to eliminate organic additives (i.e., like ligands, surfactants, thickeners) during deposition of CZTS precursors. As a direct consequence, the possibility of formation of a carbon-rich fine grained layer between the active layer and the Mo substrate is reduced thus decreasing the level of impurities known to be detrimental for minority carriers in photovoltaic devices^11^. Starting from this result, the selenization process of as-deposited CZTS has been split in two steps. The first step annealing was performed on a simple hot plate in a N2 glovebox with the following temperature profile: a 2 min ramp from room temperature to 300 °C followed by a 15 min dwell at 300 °C, and finally a natural cooling down. After this first annealing step, the samples were placed in a graphite box covered with a lid and containing 60 mg of Se and 120 mg of SnS for selenization. The thermal profile of the second step annealing is designed to follow: a ramp of 30 °C/min to 320 °C, a 2 min dwell at 320 °C, a second ramp of 12 °C/min to 550 °C followed by a 25 min dwell at 550 °C and a natural cooling. The 60 mg Se mass in the graphite box was tailored to reach our current optimum chalcogens ratio ([S]/[S+Se]) content in the material. The presence of the 120 mg SnS pellet is useful to diminish the possible tin-related losses, which could take place during the selenization annealing process.

### Device fabrication

After selenization, the heterojunction was completed with approximately 50 nm of CdS layer deposited on top of CZTSSe absorber using chemical bath deposition (CBD) method. On top of CdS, a bilayer composed of i-ZnO (50 nm)/Al:ZnO (450 nm) was sputtered as the window layer. Intrinsic ZnO was deposited using the same condition as mentioned before for 15 mins. Al:ZnO is deposited by RF-sputtering (HHV 500 Auto vacuum coater, 1.1 W/cm^2^, 10^−3 ^mbar, 6 sccm Ar, room temperature, 90 mins), using 99.99% purity targets. Finally, on top of Al:ZnO, a patterned Ni/Al layer was thermally evaporated as a front electrode. The dimension of individual solar cells was 4 mm^2^.

### Characterization

The CZTSSe alloy structures were examined by powder X-ray dispersion (XRD) and Raman spectroscopy. XRD experiments were performed using Cu-K alpha radiation (tube voltage 40 kV and tube current 40 mA). The diffraction pattern was obtained with a step width of 0.015. Raman measurement was carried out using Horiba Jobin-Yvon LabRam spectrometer with green laser (514 nm) excitation. The surface morphology of the films and its composition were evaluated by FEI XL-30 scanning electron microscopy (SEM) equipped with energy dispersive X-ray spectroscopy. Solar cells were measured under AM1.5 simulated solar light with intensity of 100 mW/cm^2^ using an Oriel Solar 1A. External quantum efficiency (EQE) measurements are carried out in a Lot Oriel Spequest RERA with a monochromator under chopped illumination and a lock-in technique. A Horiba glow discharge spectroscopy (GDS) tool was used to evaluate the elemental depth profile.

## Results & Discussion

### Current-voltage characteristics

**Figure 1:**
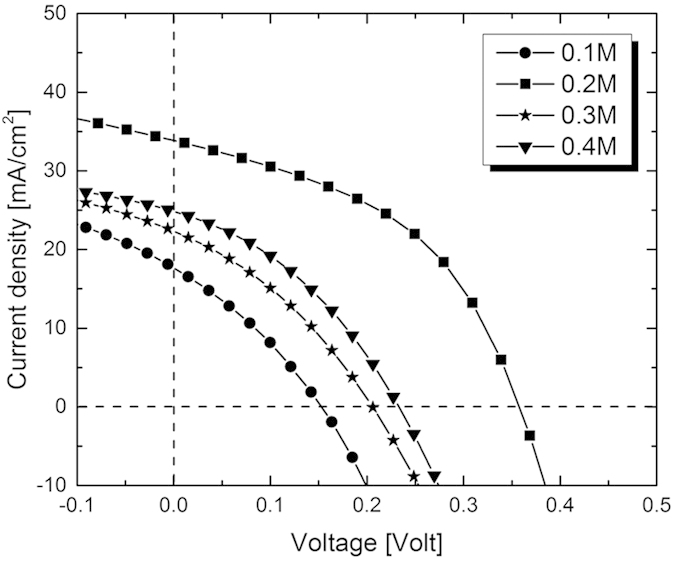
J–V characteristics of the best-performing Mo/CZTSSe/CdS/TCO/Ni/Al solar cell based on CZTS treated with different sodium concentrations.

Sodium has been demonstrated to be the most useful element to enhance PV performances of CZTSSe. Na incorporation was optimized in ESAVD-deposited kesterites in this work. To do that, four CZTS precursor layers dipped in aqueous sodium solutions with different concentrations (in molar concentration): 0.1, 0.2, 0.3, and 0.4 M for 20 minutes have been employed in full PV devices. A statistical study has been performed on more than 36 solar cells for each sample. The J–V characteristics derived from the best performing photovoltaic devices are depicted in Fig. 1. It is demonstrated that a sodium concentration of 0.2 M was required to achieve the best power conversion efficiency of 5.5% with Voc of 355 mV, Jsc of 33.5 mA/cm^2^, and a FF of 47.8%. This result confirms what has already been demonstrated for CZTSSe deposited via vacuum technology and other non-vacuum technologies: the PV performances of the solar cells increase along with Na concentration until an optimum point before decreasing.

**Figure 2:**
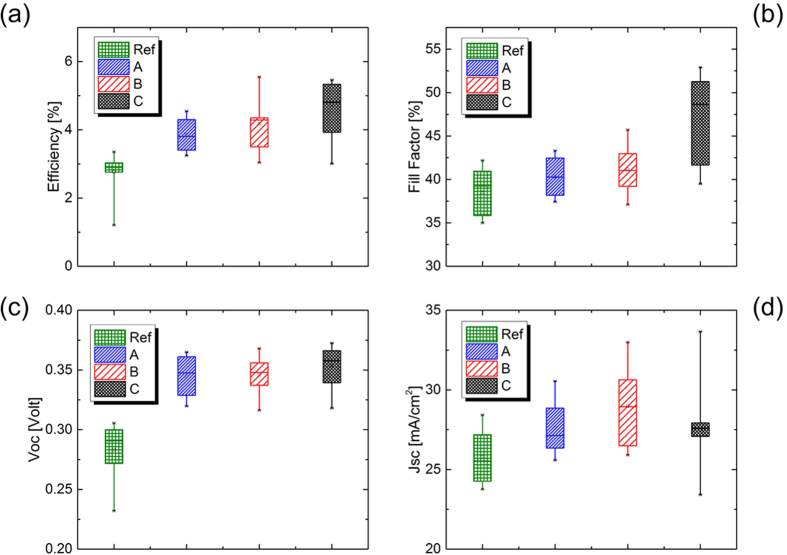
PV parameters of CZTS solar cells incorporated with different alkali ions before thiourea treatment.

Based on the results shown above, other alkali metals (Li, Rb) were also incorporated in CZTSSe layers and their influence on the PV performances of the solar cells was studied. The element incorporation condition was the same as that used for the best sodium-doped solar cell, 0.2 M diluted solution, and was kept fixed from this point on. Again a statistical study has been performed on more 36 solar cell for each sample to verify the robustness of our assumptions and conclusions. Each sample was further compared with a reference sample for which no doping was applied. Figure 2 shows PV parameters of CZTSSe solar cells incorporated with different alkali ions.

The median power conversion efficiency in the case of all doped samples was higher than the reference sample (as-produced sample, without exposure to alkali ions) showing the beneficial effect of alkali ion incorporation. Median PCE of sample C (4.4%) solar cells was higher than sample A (3.9%) and B solar cells (4.1%) (Fig. 2a). The variation of PCE between samples was mainly dominated by the variation of the fill factor: 40.5% (sample A), 41.8% (sample B), 48.2% (sample C) (Fig. 2b). The Voc offered a more homogeneous trend: 0.35 V (sample A), 0.35 V (sample B), and 0.37 V (sample C) (Fig. 2c). These results confirmed, as for other techniques used for kesterite deposition, that alkali elements can boost PV performances in ESAVD-deposited CZTSSe solar cells.

Once the most adapted alkali molar concentration (0.2 M) was established, we varied the exposure time from 20–60 mins to further optimize the alkali ion incorporation conditions. The efficiencies of best-performing CZTSSe solar cells doped with Li, Rb and Na for different exposure time are summarized in the Table 2.

**Figure 3:**
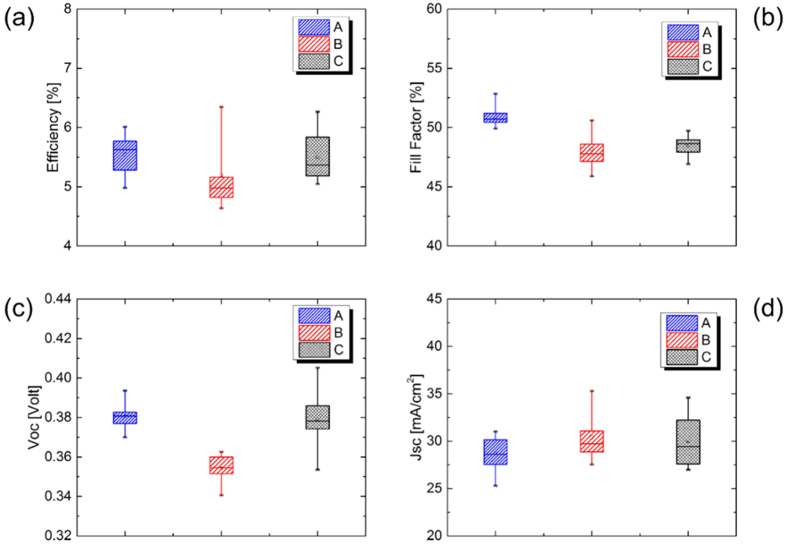
PV parameters and characteristics of the best-performing Mo/CZTSSe/CdS/TCO/Ni/Al solar cells exposed to alkali elements (A = Li, B = Rb and C = Na) after thiourea treatment.

The results showed that further incorporation (after 20 minutes) of doping agents (non-quantified in the absorber) did not improve PV performances of CZTSSe solar cells. Based on these results, we have fixed soaking time of 20 minutes in 0.2 M of alkali solution for CZTSSe precursor before selenization. The next step is to try to improve CZTSSe/CdS interface by using combination of HCl and thiourea treatment of selenized absorber before buffer layer deposition. Details on this techniques were described in ref. 12. A statistical study of light PV characteristics was performed on more than 25 cells with different alkali contents. The results are reported in Fig. 3. The median power conversion efficiency of Rb-doped CZTSSe solar cells was lower (4.9%, see Fig. 3a) as compared to the other two type of solar cells (Li 5.7% and Na 5.5%). The variation of PCE between cells was due to the variation of the median fill factor (Fig. 3b) and Voc (Fig. 3c). Possible process variance during CdS and TCO deposition could also lead to these differences in FF between the three types of CZTSSe solar cell as demonstrated by EQE later in this paper. Median Voc for Li and Na-doped CZTSSe (0.38 V) was higher than Rb-doped CZTSSe (0.35 V), however, the Jsc appears to be comparable for Rb and Na-doped CZTSSe (29.2 mA/cm^2^) and lower for Li-doped CZTSSe (27.1 mA/cm^2^).

From this study, we have established that the HCl+ thiourea treatment has enhanced the PV performances of our solar cells in accordance to ref. 12. As suggested by Furuta *et al.* the improvement of the characteristics seems to be due to improvement of the film quality and increase in the width of a depletion layer^12^. The latter assumption is demonstrated by the augmentation of the short-circuit current in the comparison between the same solar cells with and without HCl and thiourea treatment (Figs 2 and 3).

**Figure 4:**
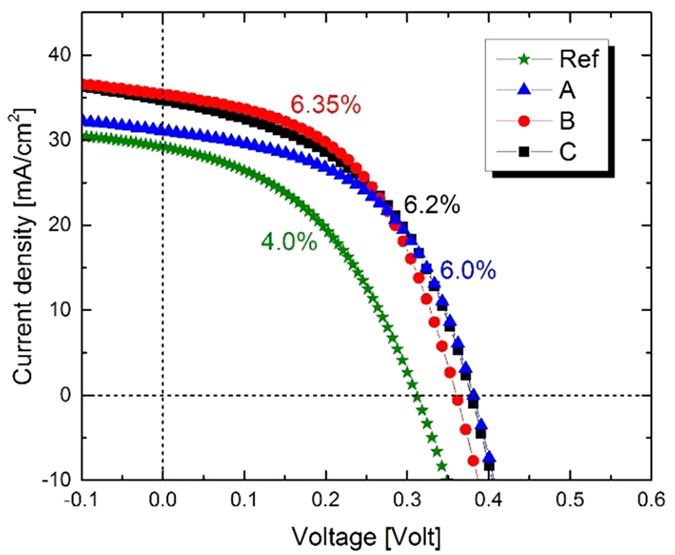
J–V characteristics of the best-performing Mo/CZTSSe/CdS/TCO/Ni/Al solar cells for samples with best-performing alkali elements (A = Li, B = Rb and C = Na) after thiourea treatment.

The PV performances of our best CZTSSe solar cell for the three types of doping were similar and the related J–V curves are shown in Fig. 4. In particular the highest efficiency was reached by Rb-doping (sample B). The current-voltage characteristics show a solar cell with 6.35% efficiency, 49.9% FF, 360 mV Voc and a Jsc of 35.2 mA/cm^2^. The best efficiency for Li (sample A) and Na-doped CZTSSe (sample C) PV devices are 6.0% and 6.2%, respectively, and the projection of the other PV parameters followed the trend reported above in the statistical study.

### External quantum efficiency

**Figure 5:**
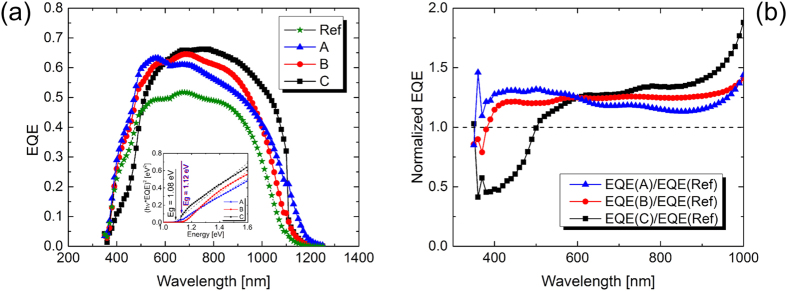
External quantum efficiency measurements on best performing CZTSSe solar cells with different alkali ions incorporation (**a**). EQE spectrum ofCZTSSe solar cells with different alkali ions incorporation divided by the EQE spectrum of a reference undoped solar cell (**b**). The inset in Figure 5a is the bandgap Eg deduced via linear extrapolation of the low energy slope of the EQE.

In order to study the spectra response of solar cells using alkali ions incorporated absorbers, EQE measurements on the best performing CZTSSe solar cells containing Li–, Na– and Rb have been carried out and the related results are shown in Fig. 5a. A solar cell with no alkali ion treatment was used as a reference sample, although a small amount of alkali elements from the soda-lime glass substrate might have been migrated into the absorber during selenization. The quantum efficiency in the visible range is promising (~50%) for the three cells; the blue response is reduced in the case of CZTSSe-Na probably due to front surface recombination. The three curves have higher EQE in the near-infrared range (750 nm–1200 nm) as compared to standard CZTSSe solar cells with no alkali content. This could be mainly due to the result of a better current collection due to higher diffusion length of minority carriers generated deep in the absorber (Fig. 5b). The extracted optical bandgap is around 1.08 ± 0.02 eV for CZTSSe-Li and CZTSSe-Rb, whereas a bandgap of 1.12 ± 0.02 eV is extracted for CZTSSe with Na-content (inset in Fig. 5a).

### X-ray diffraction

**Figure 6:**
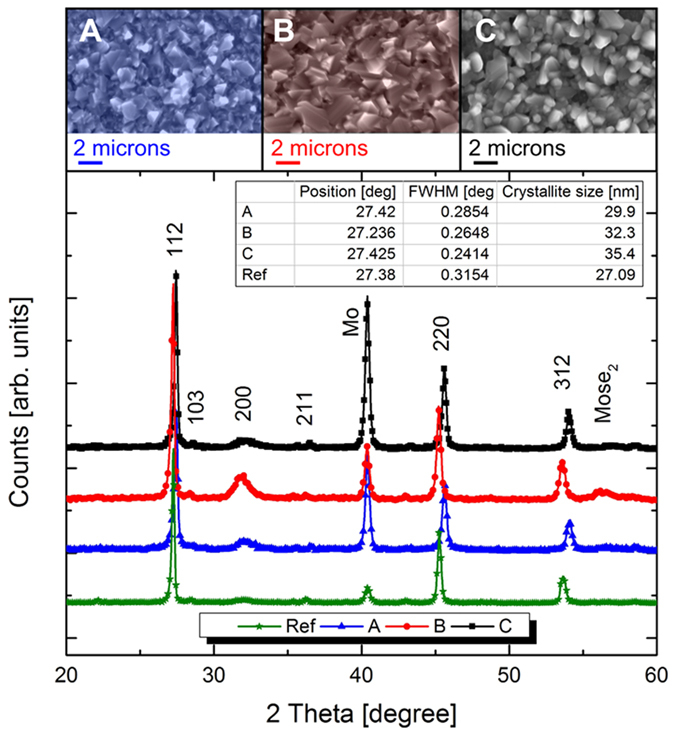
X-ray diffraction and relative cross-sectional SEM images of CZTSSe after selenization for samples A (**a**), B (**b**) and C (**c**). The inset contains the crystallite size of the different samples extracted from the 112 diffraction peak.

The XRD reflections of CZTSSe alloys at room temperature are shown in Fig. 6. The XRD patterns of all samples exhibit major peaks corresponding to diffraction lines of the kesterite structure of CZTSSe^13^. No distinct peaks of secondary phases were observed in the XRD pattern apart from a MoSe^2^ reflection at 56.2°. The diffraction peaks from (112), (200), and (312) planes were observed clearly in all samples indicating the formation of well crystallized CZTSSe phase. The presence of a single and symmetric (112) diffraction peak in all measured diffractograms indicates that all samples are homogeneously alloyed rather than a mixture of CZTS and CZTSe phases. Moreover, the shift in peak position towards higher angles for sample A and B was due to the higher [S]/[S/Se] ratio as shown in EDX. XRD has also been used to estimate the crystallite size of CZTSSe by using Scherrer’s equation^14^. The calculated crystallite dimension of the three samples was calculated and compared to the one of a reference absorber with no doping (27.09 nm): the results, exposed in the inset of Fig. 6, showed that the highest crystallite size was achieved in the case of sample B (35.4 nm), whereas for sample A and C the mean crystallite dimension was 29.9 and 32.3 nm, respectively. This showed that there is an increase in the size when the CZTSSe layer contains higher Na-content as confirmed by Prabhakar *et al.*^14^. Considering that a typical crystallite size on a reference ESAVD-synthesized CZTSSe is circa. 24.2 nm^6^, XRD results indicated that Li, Rb and Na ion incorporation could increase the crystallite size of CZTSSe absorber thus decreasing the possibility of charge recombination at the grain boundaries.

In order to verify these findings, the SEM images of the surface morphology of post selenized thin films are shown in Fig. 6. The surface images of the three samples exhibited dense and well-grown crystalline texture with noticeable grains as a consequence of selenization. The same trend as for crystallite size was found for grain size of the three samples. The grain size measurement showed that bigger grains were achieved for sample B (larger than 2 microns), slightly smaller ones were visible for sample A and C.

### Energy-Dispersive X-ray Spectroscopy

Energy dispersion X-ray measurements, in top-view configuration at 25 kV was performed to evaluate compositional changes in the selenized absorber layers and the results are summarized in Table 3. The evaluation of the metal ratios [Zn]/[Sn] and [Cu]/[Zn+Sn] in CZTSSe layers is a critical point since improved performance in solar cells has been attributed to a Cu-poor and Zn-rich absorber layer.

The [Cu]/[Zn+Sn] ratio was similar for all samples (0.83 / 0.81 / 0.85) whereas [Zn]/[Sn] ratio was higher for sample A (1.15) than samples B (1.11) and C (1.10). This was mainly due to Sn-losses in sample A, which typically occurs during selenization^9^. An average alloy composition of around x = 0.14 ([S]/[S+Se]) for sample A and B also indicated that Se replaced S in the absorber during the annealing, whereas an alloy x = 0.07 for sample B indicated that almost no S was left in the absorber, therefore, a pure CZTSe layer might have left behind after selenization (the results are summarized in Table 3). Chalcogens ratio was consistent with the compositions obtained from the EQE measurements calculated based on the Vergard’s law applied on the bandgap energies which were determined from EQE measurements of solar cell devices based on these films. Anion concentration obtained from EDX was also in good agreement with the XRD measurements.

### Raman spectroscopy

**Figure 7:**
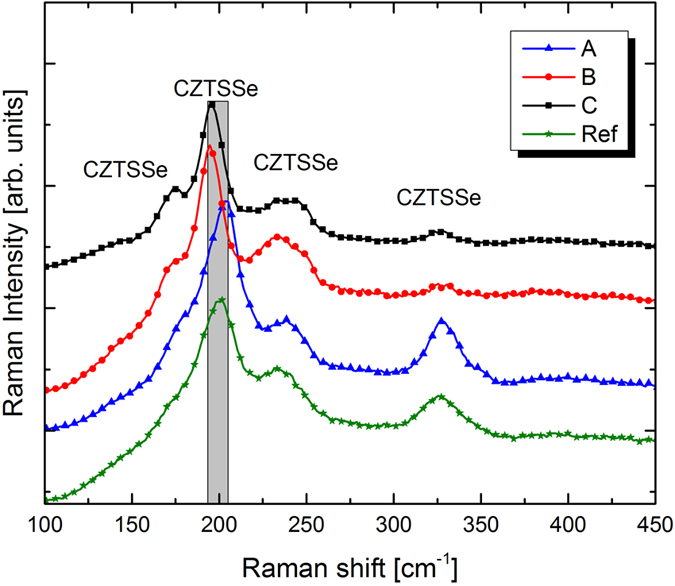
Raman spectra of selenized CZTSSe absorber for sample A, B and C.

The possible presence of minor phases was characterized by Raman spectroscopy. CZTSSe compounds in their kesterite structure (space group I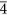) leads to the theoretical prediction of 27 active Raman modes^15^. Figure 7 shows the Raman spectra of samples A, B and C. Two dominant Raman peaks were observed in this system and assigned to A symmetry modes: CZTSe-like (165–205 cm^−1^) and CZTS-like (280–350 cm^−1^) in agreement with the bimodal behavior as described in ref. 16. Since Raman scattering is a surface sensitive technique, the shift of the CZTSSe peaks, as a function of the chalcogens ratio, is only meaningful for approximately the first 100 nm of the layer (penetration depth in CZTSSe with green excitation). However, these results were consistent with the overall anion compositions obtained from EDX, EQE and XRD measurements.

The Raman spectra from the three samples also showed additional CZTSSe peaks in the frequency region (220–260 cm^−1^)^15^. The observed additional peaks could not be attributed to secondary phases: (i) no ZnSe secondary phases has been detected under blue excitation wavelengths, which are resonant under these conditions^17^, (ii) no ZnS reflections^18^ appears in XRD diffractions, (iii) no spectral contributions from Sn(S,Se) and Cu_x_(S,Se) phases are expected from the XRD results (Sn-rich phases) and synthesis conditions (Cu-rich phases). As the cationic composition ([Cu]/[Zn+Sn] and [Zn]/[Sn]) resulted almost equivalent for the three samples from EDX analysis, it is expected that changes in Raman peak positions was caused mainly by the anion composition variation ([S]/[Se+S]). The latter assumption is proved by the variation of the A1 CZTSe and CZTS peaks in Fig. 7. Furthermore, full-width-at-half-maximum (FWHM) of peaks was the same for the most intense CZTSSe peak (196–205 cm^−1^) of samples A, B and C. This indicated the absence of significant chemical disorder variation compared to reference CZTSSe with no alkali doping^15^. The lower FWHM for the CZTSSe peak in the case of reference cell, suggested the possible improvement of the quality of the absorber, due to alkali incorporation, without modifying the composition of the latter.

### Glow Discharge Spectroscopy

**Figure 8:**
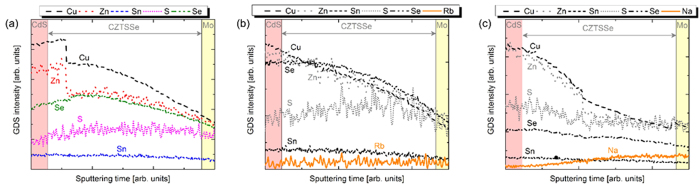
GDS spectra of selenized CZTSSe absorber for reference sample with no doping (**a**), sample B (**b**) and C (**c**).

Glow discharge spectroscopy was used for real-time detection of the different elements^1^; the surface roughness of each sample could give rise to some noise in the first part of the GDS spectra, but this does not influence the conclusions of the present study. The GDS spectra of a reference sample with no doping, sample B and C can be seen in Fig. 8.

The CZTSSe GDS spectrum of a reference sample with no alkali incorporation is depicted in Fig. 8a. The elements profile show that S, Se and Sn are constant along the thickness of the absorber, whereas Cu and Zn concentration increases towards the surface of the CZTSSe layer. The same situation is visible in the case of Rb- (sample B) and Na- (sample C) incorporation in the absorber layer (Fig. 6b,c) as well as for sample A (not shown here). The only remarkable difference in between the three samples is the fact that, the Se profile dramatically increase from the back contact towards the CdS layer in sample B: this high concentration of Se on the surface of the sample is in good agreement with the Raman analysis. The latter assumption is corroborated by a stronger CZTSe peak (196 cm^−1^) detected due to higher Se concentration on the surface of the CZTSSe (Fig. 7) considering that Raman spectra derived from green light excitation can probe only the first 100 nm of the CZTSSe sample.

## Discussion

The experimental results show that, the incorporation of Li, Rb and Na can improve photovoltaic properties of CZTSSe solar cells. As showed by XRD and Raman, the authors speculate that alkali elements improved the quality of the absorber by: (i) depleting it from the appearance of harmful secondary phases; (ii) increasing the crystallite size thus reducing carrier recombination near the boundaries; (iii) the absence of significant chemical disorder effects. The increase of the quality of the absorber was also testified by the increase of the collection efficiency of the minority carriers as demonstrated by EQE in the near-infrared range. Moreover, GDS analysis has shown that Li, Rb and Na have no apparent influence on the the uniformity composition of the compound when compared to a reference CZTSSe with no alkali incorporation.

## Conclusion

For the first time the effects of incorporation of different doping elements (Li, Rb and Na) on the properties of CZTSSe-based solar cells deposited by ESAVD have been studied. These doping materials were incorporated in the CZTSSe compounds by exposing the CZTS precursor samples in a solution containing these elements prior to selenization annealing. The molarity of the solution and the exposure time have been varied to establish the optimum conditions: 0.2 M for 20 minutes. A statistical study of the PV performances of the derived solar cells has been performed and the results show that the incorporation of Li, Rb and Na are beneficial for PV performances since they can improve the quality of the absorber by increasing grain size and prevent the formation of armful secondary phases. The reported efficiency for the best performing solar cells is 4.8%, 5.7% and 5.5%, respectively for Li-, Rb- and Na-incorporated CZTSSe. Further gain in PCE is reached by performing an HCl+ thiourea treatment after the selenization process and before CdS deposition. This step led the overall efficiency to 6.0% for Li, 6.2% for Na, and 6.35% for Rb, which is currently the best reported result for ESAVD-deposited CZTSSe solar cells.

## Additional Information

**How to cite this article**: Altamura, G. *et al.* Influence of alkali metals (Na, Li, Rb) on the performance of electrostatic spray-assisted vapor deposited Cu_2_ZnSn(S,Se)_4_ solar cells. *Sci. Rep.* **6**, 22109; doi: 10.1038/srep22109 (2016).

## Figures and Tables

**Table 1:** Association of sample name with incorporated metal in CZTS precursor.

Sample name	A	B	C
Incorporated metal	Li	Rb	Na

**Table 2:** Efficiencies of the best-performing CZTS solar cells doped with Li, Rb and Na for different ducking time (20–40–60 mins).

Sample Name	A			B			C		
Exposure time mins	20	40	60	20	40	60	20	40	60
Average PCE %	4.5	4.2	4.4	5.7	5.2	5.0	5.5	4.8	4.4

**Table 3:** Elemental composition ratios of samples A, B and C.

	A	B	C
[Cu]/[Zn+Sn]	0.83	0.81	0.85
[Zn]/[Sn]	1.15	1.11	1.10
[S]/[S+Se]	0.16	0.07	0.12

Results were obtained form top-view EDX measurements.
